# Adapting to the Future: Introducing the Radiation Oncology Alliance Adaptive Radiotherapy Working Group

**DOI:** 10.1111/1754-9485.70023

**Published:** 2025-09-16

**Authors:** Meegan Shepherd, Kenton Thompson, Trent Aland, Jeremy Booth, Jeremiah de Leon, Cathy Hargrave, Michael G. Jameson, Sweet Ping Ng, Amy Yuen Meei Teh, Nigel Anderson

**Affiliations:** ^1^ Northern Sydney Cancer Centre Royal North Shore Hospital St Leonards New South Wales Australia; ^2^ Monash University Clayton Victoria Australia; ^3^ Peter MacCallum Cancer Centre Melbourne Victoria Australia; ^4^ Sir Peter MacCallum Department of Oncology The University of Melbourne Melbourne Victoria Australia; ^5^ Icon Group Brisbane Queensland Australia; ^6^ Faculty of Science Queensland University of Technology Brisbane Queensland Australia; ^7^ Institute of Medical Physics, School of Physics University of Sydney Sydney New South Wales Australia; ^8^ GenesisCare Sydney New South Wales Australia; ^9^ Radiation Oncology Princess Alexandra Hospital—Raymond Tce Campus Brisbane Australia; ^10^ Faculty of Health, School of Clinical Sciences, Queensland University of Technology Brisbane Queensland Australia; ^11^ University of New South Wales Sydney New South Wales Australia; ^12^ Centre for Medical Radiation Physics University of Wollongong Wollongong New South Wales Australia; ^13^ Olivia Newton‐John Cancer Wellness and Research Centre Austin Health Heidelberg Victoria Australia; ^14^ University of Melbourne Melbourne Victoria Australia; ^15^ Icon Cancer Centre Wahroonga New South Wales Australia; ^16^ Sydney Adventist Hospital Wahroonga New South Wales Australia; ^17^ ANU College of Health and Medicine The Australian National University Canberra Australian Capital Territory Australia; ^18^ Australian Society of Medical Imaging and Radiation Therapy Melbourne Australia; ^19^ Department of Medical Imaging and Radiation Sciences, Faculty of Medicine, Nursing and Health Sciences Monash University Clayton Victoria Australia

**Keywords:** quality assurance, radiation oncology, radiation oncology imaging

## Abstract

This paper details the current state of Online Adaptive Radiation Therapy (oART) in Australia and New Zealand (ANZ) and identifies the need for best practice guidelines for ANZ. This paper has been formulated by a working party formed as a branch of the Radiation Oncology Alliance (ROA), which is the peak group comprising the organisations that represent the four key professions in radiation oncology—the Royal Australian and New Zealand College of Radiologists (RANZCR), the Australasian College of Physical Scientists and Engineers in Medicine (ACPSEM), the Australian Society of Medical Imaging and Radiation Therapy (ASMIRT) and the Cancer Nurses Society of Australia (CNSA). The ROA Adaptive Radiation Therapy working party consists of membership from RANZCR, ACPSEM and ASMIRT.

## 1 Background

Yan et al. [1] first proposed the concept of adaptive radiation therapy (ART) in the 1990s. They described ART as a process of modifying treatment plans via a systematic process of measuring feedback. Since then, technological advancements have enabled more sophisticated and efficient planning tools, allowing for rapid online replanning, accounting for patient anatomical and functional changes during treatment [2]. Clinical and economic benefits are emerging and evolving as technological improvements are introduced and may reduce apparent costs [3, 4]. Online adaptive radiation therapy (oART) is rapidly gaining clinical uptake in ANZ using specialised linacs. The first oART capable linacs in ANZ were implemented in 2019 and now we have 8 systems treating the full range of indications.

## 2 Definitions

The following are the definitions of ART used in this paper, in order to differentiate between the nuances of each and the environment in which they are applicable.

## 3 Offline ART

Offline ART is the process of adjusting the patient's treatment plan parameters based on observed, systematic changes that are modified after the current, delivered treatment fraction [5]. The replanning typically follows the same clinical workflow as regular initial treatment planning to adapt to systematic changes, for example, patient weight loss, tumour response or morphological changes or ‘plan of the day’ approaches [6]. Offline ART is generally applied using conventional replanning and conventional linacs, however the tools applied to oART are emerging to streamline processes offline.

## 4 Online ART

Online ART is a process where the patient's treatment plan is adjusted prior to the daily treatment delivery to account for variations detected, while the patient remains in the treatment position [1, 7].

It allows the radiation plan to be adjusted to information of the day (i.e., anatomy or functional imaging) [8], with the new plan created immediately before beam delivery. Due to the computational requirements and truncation of multiple workflow processes, this is generally limited to purpose‐built oART linacs. There are also emerging cloud‐based software tools that could revolutionise oART for conventional linacs.

## 5 Real‐Time ART

Real‐time ART is a process of adapting to account for continuous variations that occur within a treatment fraction, for example, respiration, internal status changes, peristalsis motion and tumour morphological changes. It is an emerging field and allows for changes during the delivery of a treatment fraction, for example, multi‐leaf collimator (MLC) tracking [9].

## 6 Current oART Platforms Available in Australia and New Zealand

The principal purpose of online adaptation is to enhance the accuracy of daily radiotherapy treatment delivery. Current platforms available in ANZ use either magnetic resonance imaging (MRI)‐guided or cone beam computed tomography (CBCT)‐guided linacs [10, 11]. Both these technologies allow for full customisation, creation, and delivery of new or partially optimised treatment plans.

## 7 Potential Benefits of oART: Literature Available To‐Date

Research has demonstrated that oART can conform radiation dosimetry to the anatomy of the day, allowing margin reduction that can reduce doses to organs at risk (OAR) and potentially decrease radiotherapy associated toxicities. Improved conformality equally allows ultra‐hypo fractionation, increased prescription doses or simply improves target dose delivery. This provides the possibility of enhanced local control, overall survival and improvement in other patient outcomes [8]. The benefits of oART technology have been observed across various body sites, both for radical and palliative treatments, including head and neck [12, 13], lung [14], abdominal [15] and pelvic cancers [10, 16].

## 8 State of oART Technology and Its Adoption in Australia and New Zealand

Both CBCT and MRI guided adaptive linacs were installed in ANZ in 2019. Industry research collaborations, such as those with Varian Medical Systems (Palo Alto, USA) and Elekta (Stockholm, Sweden), have initially driven advancements in oART due to high costs. Significant progress is evident at leading institutions like Northern Sydney Cancer Centre (NSCC), Olivia Newton‐John Cancer Wellness and Research Centre (ONJCWRC), Townsville Hospital, GenesisCare (GC) Oncology at St Vincent's Hospital and Murdoch and Icon Cancer Care where oART platforms are clinically available. These centres are at the forefront of oART implementation, clinical application and research [10, 11, 17, 18, 19, 20, 21, 22, 23, 24, 25, 26] with multidisciplinary teams mirroring conventional treatment planning methods. Over the next 5 to 10 years, the expected increase in oART‐capable linacs will integrate oART into routine clinical practice, enhancing patient outcomes across the region.

## 9 Challenges in Implementing and Adopting oART

Despite oART advancements, challenges persist, particularly in accessibility, time taken, resources, and the need for multidisciplinary team involvement in daily adaptive sessions [17, 18]. Efforts are underway to address these issues through significant investments in professional development and collaborative initiatives by ASMIRT, RANCZR and ACPSEM. These initiatives focus on ensuring patient safety, quality assurance and effective use of adaptive technologies. Emphasis is placed on comprehensive education and training to develop a skilled workforce capable of maximising oART benefits. By overcoming these challenges through targeted collaborations, improved access to oART and enhanced patient outcomes across ANZ are anticipated.

## 10 State of oART Technology: Global Adoption

Looking globally, the status of oART provides valuable insights for creating guidelines in ANZ. Regions like North America, the United Kingdom and European nations like Denmark and the Netherlands have developed diverse and innovative approaches to oART, reflecting unique workforce structures and healthcare professional training. Globally, there has been proactive integration of oART into clinical practice, driven by research initiatives in feasibility, safety and collaboration among leading oncology centres and professional bodies in radiation therapy. The ‘R‐IDEAL’ framework has been proposed for systematically evaluating RT technology to ensure proven effectiveness and preventing unnecessary harmful patient exposures [27].

Our oART community is currently leveraging oART technology to advance local and international collaborations in research on patient suitability, clinical benefits and innovation. Clinical research studies continue to build on demonstrating enhancements in patient outcomes across various treatment indications, including improved treatment efficacy and reduced toxicity [4].

## 11 Artificial Intelligence and oART

The success of oART technologies depends on automation of processes and will incorporate artificial intelligence (AI) to enhance efficiency. While AI can significantly optimise oART workflow, human oversight is essential [28]. Training and credentialing must ensure that users understand both the capabilities and limitations of AI, including potential risks. Understanding AI algorithms, including their data dependencies and inherent biases, is crucial for safe and effective use in clinical settings [29].

## 12 The ROA Working Party

The creation of the ROA Working Party, with representatives experienced in oART from RANCZR, ACPSEM and ASMIRT, marks the initiation of a best practices guideline for oART in ANZ. This collaborative effort ensures the development of comprehensive and practical guidelines that address the unique challenges and needs of the local Radiation Oncology community. By drawing on the expertise of local users and professionals, the guidelines will promote a unified approach to oART, prioritising patient safety and clinical excellence specific to ANZ's working environment. Additionally, the guidelines aim to address gaps identified in global literature and ensure that ANZ departments can implement standardised procedures and workflows effectively, recognising that there will be a need for these guidelines to be reviewed regularly to ensure currency with clinical practice. This initiative will improve cancer patient outcomes by fostering consistent, high‐quality care across the region and positioning ANZ as a leader in oART best practices.

## 13 ROA Working Party Stakeholders

– RANCZR: Dr. Sweet Ping NG (ONJCWRC), Dr. Amy Teh (Icon Care), Dr. Jeremy de Leon (GC).
– ASMIRT: Meegan Shepherd (Chair; NSCC), Dr. Nigel Anderson (ONJCWRC & ASMIRT Executive), Dr. Cathy Hargrave (QLD Health/QUT), Kenton Thompson (Peter Mac), Min Ku (Secretary; ASMIRT support).
– ACPSEM: A/Prof Michael Jameson (GC), Prof Jeremy Booth (NSCC), Dr. Trent Aland (ICON Care).

## 14 Components of the Guidelines

Establishing guidelines for oART implementation and safe clinical practice is essential for ensuring consistent, high‐quality care across diverse clinical settings. These guidelines will address critical aspects including clinical and economic benefit, applications, workflow, staffing, training, credentialing, scope of practice, QA and patient care standards, creating a unified framework for oART delivery. Clear inclusion and exclusion criteria for patient selection are crucial to optimise outcomes and allocate staffing effectively. Key practice considerations will include factors such as patient preparation (e.g., bladder filling), margin reduction strategies, dose fractionation, dose accumulation and QA measures. These are integral to enhancing the precision and effectiveness of oART treatments. Departmental planning, including staffing models and streamlined workflows, is necessary to integrate oART seamlessly into clinical practice. Pre‐planning strategies, treatment templates and real‐time plan adjustments should be incorporated to maximise efficiency and minimise delays. Comprehensive and continuous training programmes are essential to ensure all staff are proficient in the latest oART techniques and emerging technologies, such as AI and multimodal imaging. Ongoing QA practices should be built into the system to assess and enhance the quality of oART procedures, ensuring they meet the highest standards of patient care. Clinical case studies and trials demonstrating effective pre‐planning, contouring workflows and treatment efficiencies will provide invaluable insights for radiation oncology departments aiming to adopt oART. Safety protocols are also critical to protect both patients and staff, addressing potential risks during treatment.

## 15 Conclusion

oART is progressing across ANZ, with future plans for more oART linacs and upcoming clinical applications for existing C‐arm linacs. However, high costs, workflow integration issues, limitations to delegation and a shortage of trained professionals remain significant challenges. To overcome these barriers, local and international collaboration, enhanced training and standardised protocols are essential. The ROA and its ROA Adaptive Radiation Therapy working party are aiming to play a key role in establishing best practices and ensuring patient safety, with region‐specific guidelines set for release in 2025. These guidelines will incorporate local expertise to promote a unified approach to oART. By addressing current challenges and fostering collaboration, ANZ can improve cancer patient outcomes and position itself as a leader in oART practices.

## Author Contributions

**Meegan Shepherd:** conceptualization, writing – review and editing, writing – original draft, investigation, project administration, visualization. **Kenton Thompson:** conceptualization, writing – review and editing, investigation, visualization, writing – original draft. **Trent Aland:** conceptualization, writing – review and editing, investigation, visualization, writing – original draft. **Jeremy Booth:** conceptualization, writing – review and editing, investigation, visualization, funding acquisition, supervision, writing – original draft. **Jeremiah de Leon:** conceptualization, writing – review and editing, investigation, visualization, funding acquisition, supervision, writing – original draft. **Cathy Hargrave:** conceptualization, writing – review and editing, investigation, visualization, writing – original draft. **Michael G. Jameson:** conceptualization, writing – review and editing, investigation, visualization, writing – original draft. **Sweet Ping Ng:** conceptualization, writing – review and editing, investigation, visualization, writing – original draft. **Amy Yuen Meei Teh:** conceptualization, writing – review and editing, investigation, visualization, writing – original draft. **Nigel Anderson:** conceptualization, writing – review and editing, investigation, supervision, visualization, funding acquisition, writing – original draft.

## Conflicts of Interest

The authors declare no conflicts of interest.

## Data Availability

The authors have nothing to report.
